# Etude des relaparotomies précoces aux Hôpitaux Universitaires de Lubumbashi: aspects épidémiologiques, cliniques et thérapeutiques

**DOI:** 10.11604/pamj.2018.30.127.12609

**Published:** 2018-06-13

**Authors:** Catherine Saleh Ugumba, Marc Kashal Kasong, Cedrick Sangwa Milindi, Gabriel Wakunga Warach, François Tshilombo Katombe, Etienne Odimba Bfkoshe

**Affiliations:** 1 Faculté de Médecine, Département de Chirurgie, Université de Lubumbashi, République Démocratique du Congo

**Keywords:** Relaparotomie, hôpitaux peu nantis, urgences chirurgicales, Relaparotomy, hospitals with insufficient resources

## Abstract

Les relaparotomies précoces pour péritonite post opératoire (PPO) sont des urgences chirurgicales de pronostic grave. L'objectif de la présente étude est de décrire le profil épidémiologique, clinique et thérapeutique des relaparatomisés précoces soignés dans les hôpitaux universitaires de LUBUMBASHI. Il s'agit d'une étude descriptive transversale réalisée dans deux hôpitaux du district sanitaire du haut KATANGA; à savoir les cliniques Universitaires(CUL) et l'hôpital général de référence JASON SENDWE (HGR JS). Cette étude a porté sur 56 patients réopérés à l'abdomen 68 fois du 01/Janvier/2012 au 31/Décembre/2013. Ont été inclus, les dossiers des patients réopérés d'abdomen précoce deux fois de suite au moins, de sexe confondu, âgés de 07 jours à 83 ans qui ont été réopérés de l'abdomen. Les données épidémiologiques, cliniques, thérapeutiques et les suites opératoires ont été recueillies puis saisies et analysées grâce au logiciel Epi info 2011. Au cours de cette étude 304 patients avaient subi une laparotomie dont 248 avaient évolué normalement; 56 patients par contre avaient été relaparatomisés 68 fois (38 hommes et 18 femmes). L'âge moyen était de 34,6±19 ans. Le séjour moyen d'hospitalisation était de 56,26 ± 51,82 jours. Les comorbidités associées ont été l'hypertension artérielle, le cancer et le mauvais état général d'avant la laparotomie classifié ASA 3 et 4 avec respectivement 34,62% (n = 9); 26,92% (n = 7) et 41,38% (n = 12). Les malaises généraux, la circonférence abdominale avec augmentation de plus de 2 cm par jour au niveau de l'ombilic ainsi que la douleur abdominale diffuse et provoquée ont été retrouvées respectivement dans 94,64% (n = 53), 98,21% (n = 55) et 83,93% (n = 47) des cas. Pour laparotomie initiale 51 (91,07%) patients étaient opérés en urgences. Les infections associées avaient constitué l'indication principale de réintervention avec 55,36% des cas (n = 31). La laparotomie initiale été conduit par un chirurgien non qualifié dans 60,71% des cas (n = 34). Douze patients réopérés sont décédés soit un taux de létalité de 17,65%. Les relaparotomies précoces pour PPO sont couramment pratiquées dans les hôpitaux universitaires du HAUT KATANGA. Le retard dans le diagnostic aggrave le prognostic.

## Abstract (en)

Early relaparotomies due to post operative peritonitis (POP) are surgical emergencies associated with a poor prognosis. This study aimed to describe the epidemiological, clinical and therapeutic features of early relaparotomies treated at the University Hospitals in Lubumbashi. We conducted a cross-sectional, descriptive study in two Hospitals in the health district of Upper Katanga, such as the University Clinics of Lubumbashi (UCL) and the General Referral Hospitals Jason Sendwe (GRH JS). This study included 56 patients undergoing 68 abdomen operations from 01 January 2012 to 31 December 2013. The medical records of mixed-gender patients aged 07 days to 83 years undergoing early abdomen surgery at least twice were reviewed. Epidemiological clinical, therapeutic data as well as postoperative outcome data were collected, entered and then examined using Epi Info 2011. During this study, 304 patients underwent laparotomy, of whom 248 had a favorable outcome while 56 underwent 68 relaparotomies (38 men and 18 women). The average age of patients was 34.6 ± 19 years. The average length of stay in hospital was 56.26 ± 51.82 days. Relaparotomy-related comorbidities were arterial hypertension, cancer and poor physical status, classified as ASA 3 and 4 before laparotomy, accounting for 34.62% (n=9); 26.92% (n=7) and 41.38% (n=12) respectively. General malaise, abdominal circumference increased of more than 2 cm per day at the level of the umbilicus as well diffuse and induced abdominal pain were found in 94.64% (n=53), 98.21% (n=55) and 83.93% (n=47) of cases respectively. Emergency laparotomy was performed in 51 (91.07%) patients. Laparotomy-related infections were the primary indication for reoperation in 55.36% of cases (n=31). Initial laparotomy was performed by a non-qualified surgeon in 60.71% of cases (n=34). Twelve patients who underwent reoperation died, reflecting a rate of 17.65%. Early relaparotomies for POP are commonly practiced at the University Hospitals of High Katanga. Delayed diagnosis aggravates prognosis.

## Introduction

La survenue des complications précoces après laparotomies est un véritable problème de santé publique bien que sa fréquence varie d'une contré à une autre à travers le monde [1-3]. La chirurgie abdominale par laparotomie ou par laparoscopie devrait toujours être considérée comme un acte invasif et agressif susceptible de générer ses propres complications. Nonobstant, les gestes de prévention et de contrôle des facteurs de risque, cette intervention est cause de morbidité et de mortalité au premier plan aussi bien dans les pays en développement que dans les pays industrialisés [4,5]. Ces complications précoces qui font l'objet de notre étude sont celles qui apparaissent dans les 10 jours post opératoires en fonction du type de chirurgie. La survenue de la complication chirurgicale est souvent en rapport avec le type de chirurgie abdominale initialement pratiquée et la pathologie sous-jacente diagnostiquée. Ces complications postopératoires sont essentiellement: les hémorragies, les lésions ischémiques, les syndromes occlusifs et les accidents pariétaux [6-10]. La détection précoce des ces manifestations dépend d'un centre à l'autre en fonction des moyens d'investigation paracliniques disponibles susceptibles de compléter la clinique. Le but étant d'aboutir à un diagnostic rapide dans un délai raisonnable et de reintervenir en urgence afin d'améliorer le pronostic du patient. Ces explorations paracliniques sont, dans le contexte de la RD Congo, encore dérisoires et difficilement accessibles dans nos communautés aussi bien rurale et que urbaine et particulièrement en situation d'urgence. D'ou le retard de diagnostic souvent déploré et l'aggravation du pronostic vital du patient opéré.

En effet Pour certains auteurs, la survenue d'une défaillance viscérale est associée à une ré intervention tardive. Ainsi les complications post laparotomie hautement chirurgicales comme un sepsis intra péritonéal localisé ou généralisé nécessitent la précocité d'une reprise chirurgicale car les réinterventions itératives témoignent d'un problème chirurgical non résolu [11-14]. Certains auteurs considèrent la ré intervention comme un aveu fautif qu'il vaudrait mieux éviter [10,11]. Il s'avère dès lors important de détecter rapidement chez un opéré abdominal les signes cliniques et paracliniques susceptibles de conduire à une reprise précoce et améliorer le pronostic vital des patients concernés. A ce sujet les études menées à travers le monde ont démontré une grande diversité d'opinion, et nous n'avons pas trouvé dans la littérature que nous avons parcourue un protocole consensuel sur les critères précis de réintervention après chirurgie abdominale. Ces critères que certains qualifient de facteurs de risque sont en rapport avec: le patient (antécédents état général, paramètres biologiques, diagnostic étiologique), la rapidité de la décision chirurgicale, le type d'intervention pratiquée, la qualité des soins post opératoires, l'expérience du chirurgien, en RD Congo, à notre connaissance une étude exploitant ce thème, n'a jamais été initiée, raison pour laquelle nous avons mené la présente étude, qui est une étude pilote qui se limite à décrire les profils épidémiologique, clinique et thérapeutique des relaparotomies pour PPO dans les hôpitaux universitaires de LUBUMBASHI. Cette étude est préliminaire à celle qui se penchera sur les facteurs de risque de morbidité et de mortalité tels qu'observé chez les patients réopérés de PPO dans notre série de Lubumbashi en RD Congo.

## Méthodes

C'est dans deux hôpitaux universitaires de LUBUMBASHI que la présente étude a été réalisée. Il s'agit d'une étude transversale qui a été menée pendant la période qui va du 01/Janvier/2012 au 31/Décembre/2013. Au cours de l'étude nous avons reçu 304 sujets laparotomisés pour divers problèmes chirurgicaux abdominaux. 248 avaient évolué normalement et 56 s'étaient compliqués et avaient été réopérés 68 fois. Ont été inclus, tous les patients opérés d'abdomen dans les deux institutions dites (HUL) ou qui y sont transférés, et ayant subi une ou deux réinterventions pendant la période d'étude. La récolte des données était prospective à l'aide d'une fiche de récolte de données établie à cette fin et reprenant les différents paramètres en rapport avec nos objectifs.

### Les paramètres étudiés étaient

**Les aspects épidémiologiques**: (âge, sexe, institution sanitaire de gestion de la laparotomie et l'accessibilité financière aux soins médicaux).

**Les antécédents**: Avaient porté sur la comorbidité associée à la pathologie et l'état général présenté par le patient à l'opération initiale. A ces antécédents nous avons associé les caractéristiques de l'opération initiale à savoir; les circonstances de gestion de la laparotomie, les indications de la laparotomie et la qualification de l'opérateur principal.

**Les signes cliniques d'alerte**: Qui annoncent la complication à réopérer, les aspects évolutifs et le séjour hospitalier.

Signalons que plusieurs indications étaient posées par les chirurgiens et l'abord abdominal était fonction de l'acte à poser. Pour les reinterventions, la voie d'abord était exclusivement médiane sus et sous ombilicale et l'anesthésie seulement générale. Nos données ont été saisies et analysées grâce au logiciel Epi info 2011. La moyenne arithmétique, l'écart type, le mode et la médiane ont été calculés à partir des données obtenues qui sont présentées sous forme de phrases et de tableaux.

**Considérations éthiques**: Avant la récolte des données les autorisations requises étaient obtenues. Notre protocole de recherche était présenté et défendu au service de chirurgie et aussi au près du comité d'éthique de l'UNILU et cette autorisation du comité d'éthique porte le n°: UNILU/CEM/032/2014. Le consentement éclairé verbal était obtenu pour les patients de notre série. Le consentement éclairé pour les mineurs était donné par le responsable. Les informations relatives aux patients ont été tenues secrètes.

## Résultats

**L'incidence hospitalière**: Du 01 Janvier 2012 au 31 Décembre 2013; 304 opérés abdominaux étaient suivis aux (HUL) pour des circonstances et pathologies abdominales variées. 248 ont évolués normalement et ont quitté l'hôpital dans le délai jugé correct après une laparotomie. 56 patients avaient nécessité 68 relaparotomies pour des complications chirurgicales précoces soit 18,48% par rapport au total des opérés abdominaux. La fréquence hospitalière de la relaparotomie a ainsi été dans cette série de 22,37% des cas. Ceci sous-entend que toutes les 5 opérations abdominales effectuées avaient conduit à une complication réopérée.

### Aspects épidémiologiques

**De l'âge des réopérés abdominaux**: Il y a eu plus des réopérés abdominaux dans les tranches d'âge de 26 à 65 ans et de 15 à 25 ans avec respectivement 23 et 15 cas sur 56 soit 41,07% et 26,79% des cas. Les relaparotomies se font rares aux âges extrêmes de la vie. L'âge moyen de relaparatomisés était de 34,6 ± 19 ans, la médiane et le mode de 35 et 33 ans. Le patient le plus jeune était âgé de 7 jours et le plus âgé avait 83 ans. Les moins de 5 ans ont eu l'âge qui a varié entre 7 jours et 03 ans (Tableau 1, A).

**Tableau 1:** Répartition des patients relaparatomisés selon les aspects épidémiologiques

Paramètres	Effectif (n=56)	Pourcentage (%)
**Section A: age**		
-˂ 5	10	17,86
-5-14	05	8,93
-15-25	15	26,79
-26-65	23	41.07
-˃65	03	5,36
**Section B: sexe**		
-Masculin	38	67,86
-Féminin	18	32,14
**Section C: structure de gestion de la laparotomie**		
-LUBUMBASHI (HUL)	23	41,07
-Autres	18	58,93
**Section D: accessibilité financière aux soins**		
-1/3 payant de l’état (autres fonctions)	09	16,07
- 1/3 payant familial	36	64,29
-1/3 payant société	04	07,14
-1/3 payant UNILU	07	12,50

**Du sexe des réopérés abdominaux**: La prédominance du sexe masculin a été constaté chez 38 cas sur 56 soit 67,86% des cas et le sexe ratio est de 2,11 en faveur du sexe masculin (Tableau 1, B).

**De la structure de provenance de la complication réopérée**: Nous avons enregistré 33 laparotomies effectuées en dehors des hôpitaux universitaires qui étaient référées pour la prise en charge de la complication sur 56 cas soit 58,93% des cas tel que c'est illustré au tableau suivant (Tableau 1, C).

**De l'accessibilité financière aux soins**: Du point de vue financier: 36 patients sur 56 soit 64,29% des cas étaient pris en charge par la famille. Ils constituent plus de la moitié des cas tel que l'indique le tableau suivant (Tableau 1, D).

### Antécédents médicaux et caractéristiques de l'opération initiale

**Des antécédents médicaux**: Sur les 56 réopérés abdominaux 26 avaient présenté des associations morbides avant l'opération initiale. C'étaient l'hypertension artérielle, Le diabète, la diathèse hémorragique, le VIH/SIDA et la maladie cancéreuse soit 46,43% des cas le tableau 2a nous résume cette situation (Tableau 2, A).

**Tableau 2:** Répartition des patients relaparatomisés selon les antécédents médicaux et les caractéristiques l’opération initiale

Paramètres	Effectif (n)	Pourcentage (%)
**Section A: comorbidité associée**	N=26	
-HTA	09	34,62
-Diabète	06	23,08
-Diathèse hémorragique	02	07,69
-VIH/SIDA	02	07,69
-Cancer	07	26,92
**Section B: classification ASA**	N=29	
-I	05	17,24
-II	12	41,38
-III	03	10,35
-IV	09	31,03
**Section C: circonstances de gestion**	N=56	
-Urgence	51	91,07
-Programmée	05	08,93
**Section D: indications de la laparotomie**	N=56	
-Hémorragie	02	03,57
-Infection	31	55,36
-Occlusion	09	16,07
-Malformation	10	17,86
-Tumeur	04	07,14
**Section E: qualification de l’opérateur**	N=56	
-Qualifié	22	39,29
-Non qualifié	34	69,71

**De l'état général des opérés abdominaux**: Avant l'opération initiale 12 patients sur 29 soit 41,38% des cas étaient en mauvais était général. Ils étaient classés ASA III et ASA IV. Ce mauvais état général a aussi entrainé 50% et 100% des complications réopérées selon que le patient était ASA III et ASA IV le tableau suivant nous résume cette situation (Tableau 2, B)

**Du mode de gestion, de l'indication et de la qualification de l'opérateur principal de la laparotomie**: Des 56 patients compliqués, 51 soit 91,07% avaient été opérés en urgence (Tableau 2, C); Plus de 50% des cas soit 31 cas infectés sur 56 avaient constitué l'indication principale de la laparotomie. Il était ainsi suivi par les syndromes malformatifs, occlusifs et hémorragiques (Tableau 2, D); Bien que les complications réopérées des laparotomies ont pu être observées même chez les patients gérés par des mains expertes avec 39% des cas (n = 22). La majorité des laparotomies compliquées l'a été après une laparotomie initiale faite par un chirurgien non qualifié avec 61% des cas (n = 34).Le tableau suivant nous illustre cette situation (Tableau 2, E).

**Les signes cliniques d'alerte, l'évolution et le séjour hospitalier**: Plusieurs signes avaient contribué au besoin de réopérer et cela à des pourcentages variés.

**Il s'agit pour les 6 premiers respectivement de**: La circonférence abdominale qui augmente de 2cm par jour au niveau de l'ombilic 98,21% (n=55); les malaises générales 94,64% (n=53); la douleur abdominale diffuse et provoquée 83,93% (n = 47); la douleur abdominale et diffuse et permanente 82,14% (n = 46); le débit urinaire qui diminue 64,29% (n = 36) et les pansements souillés constamment (à la plaie centrale et aux drains) avec 57,14% et (n = 32) (Tableau 3, A); les aspects évolutifs et le séjour hospitalier; dans l'évolution, les défaillances circulatoires, rénales et respiratoires avaient été retrouvées chez les 12 patients réopérés abdominaux qui sont décédés. Ce qui nous donne une létalité de 21,43% par rapport aux réopérés (Tableau 3, B); pour le séjour hospitalier chez plus de 50% des patients le séjour hospitalier a été prolongé de plus de 22 jours (Tableau 3, C).

**Tableau 3:** Répartition des patients relaparatomisés selon les signes cliniques d’alerte, l’évolution et le séjour hospitalier

Paramètres	Effectif (n)	Pourcentage (%)
**Section A: signes cliniques**	N=56	
-Malaises générales	53	94,64
-respiration accélérée	16	28,57
-Circonférence à l’ombilic De plus de 2cm par jour	55	98,21
-Battements cardiaques Accélérés	19	33,93
-Douleurs abdominales Diffuse et permanente	46	82,14
-Douleurs abdominales Diffuses et provoquées	47	83,93
-Débit urinaire diminué	36	64 ,29
-Pansements souillés Constamment	32	57,14
**Section B: evolution**	N=56	
-Bonne	44	78,57
-Mauvaise	12	21,43
**Section C: séjour hospitalier (en jours)**	N=56	
≤5	12	21,43
6-10	02	03,71
11-15	05	08,29
16-21	06	10,71
≥22	31	55,36

## Discussion

**Aspects épidémiologiques** L'incidence des relaparotomies est de 22,37% et une létalité qui s'élève à 17,65%. Ceci est en contradiction avec les données anciennes de la littérature d'avant 1984 des pays nantis qui évaluaient cette incidence entre 1,5% et 3,5% après laparotomies pour péritonites postopératoires [15]. Les péritonites post laparotomies gardent théoriquement la même variation [15-17]. Ceci signe une certaine stabilité par rapport aux conditions de travail des pays nantis. Dans les pays en voie de développement et en Afrique subsaharienne, bien qu'il existe peu de données récentes dans la littérature sur cette incidence; elle est reconnue élevée et s'accompagne d'une mortalité qui avoisine les 100% quand trois défaillances viscérales sont associées ou que la prise en charge est tardive [18-21]. Cette incidence élevée chez nous serait due au retard de diagnostic des complications opératoires qui surviennent dans le contexte de chirurgie abdominale. Cette incidence peut aussi être attachée au fait que les PPO surviennent souvent chez les patients au décours d'une chirurgie réglée dans 40% des cas [22]. Ces sont les circonstances qui font le lit de la complication de l'opéré; qu'elles soient liées à l'état général de l'opéré ou aux conditions locales (chirurgie en zone cancéreuse ou en zone irradiée). A l'opposé, l'expérience du chirurgien et les difficultés techniques paraissent des éléments à prendre en compte dans l'incidence de ces infections [23]. Bien que le constat soit fait selon lequel les laparotomies se faisaient rares aux âges extrêmes de la vie, nous n'avons pas trouvé dans la littérature la prédictibilité de l'âge sur la réintervention. Certaines données confirment que le terrain parait jouer un rôle considérable dans le pronostic: l'âge avancé, les pathologies associées l'immunodépression et les défaillances d'organes.

Nous avons constaté une prédominance du sexe masculin avec 38 cas sur 56. Dans la littérature, la balance vers le sexe masculin ou féminin n'est pas retrouvée. Il est souligné par ailleurs le rôle des états morbides associés au sexe comme les anémies tropicales à prévalence plus élevée chez les jeunes femmes pendant la grossesse qui avaient une influence négative sur la cicatrisation. La provenance du patient qu'il faut réopérer a été analysée pour s'assurer si le transfert des hôpitaux de la laparotomie initiale a pu jouer un rôle dans l'évolution de la relaparotomie. 33 patients réopérés sur 56 soit 58,93% des cas étaient venus des institutions hospitalières autres que les HUL. Ces institutions hospitalières seraient situées dans des communes de la ville de LUBUMBASHI les plus peuplées, les plus étendues et sans structure sanitaire de référence. Les quelques centres de santé qui existent ne sont pas tenus par des chirurgiens qualifiés. Ils n'ont pas pour la plupart un personnel qualifié et un matériel adéquat pour le diagnostic rapide et la prise en charge adaptée. Ce qui peut expliquer les mouvements de transfert des patients compliqués vers les deux hôpitaux de référence (CUL et HGR JS) localisés en plein centre-ville. Au sein de ces hôpitaux la protection financière est assurée et aussi les conditions de prise en charge des complications des laparotomies sont plus ou moins réunies [24,25]. L'assurance des patients pour les soins médicaux n'étant pas organisée sous forme de la sécurité sociale; les patients pour être soignés en général et opérés chirurgicalement en particulier dépendent de celui et de celle ou de ceux qui payent pour eux et de la diligence de ces payeurs. La part de la famille comme support des frais médicaux a été prédominant dans la présente étude car 36 patients sur 56 soit 64,29% des cas étaient pris en charge par la famille.

Indirectement on peut déduire de ce qu'on a appelé « pauvres conditions opératoires». Les Opérations tardives etc. que cela tient aux difficultés qu'éprouvent les familles à couvrir les frais et de diagnostic et de ce qu'il est convenu d'appeler le paquet chirurgical si l'employeur n'assure pas ces frais, causant de ce fait un retard diagnostic et thérapeutique. La facilitation par la famille demandait beaucoup de temps pour que les conditions soient réunies et que la prise en charge soit amorcée. Les moins nantis donc compte tenu du faible financement des moyens diagnostiques et thérapeutiques étaient opérés tardivement. Ceci explique que leur état s'était beaucoup détérioré avant l'intervention. Une mortalité de 35% en cas de réinterventions tardives avait été rapportée [12-14]. La situation présentée au tableau II confirme les données de la littérature. Que les patients avec une comorbidité et mauvaises conditions anesthésiques posent beaucoup de problèmes pendant et après la chirurgie et en particulier les malades cancéreux. Dans les conditions de faibles ressources même les grades II de ASA donnent déjà des problèmes en raison du faible poids des moyens anesthésiques pour la réanimation. Les explorations paracliniques étaient à 100% financées par les patients qui pouvaient se prendre en charge. La facilitation qui pouvait se faire grâce au financement de la présente étude ne nous a pas permis d'inclure de manière efficace les données paracliniques qui seraient de grand apport éventuellement. Nous avons ainsi observé que la majorité des laparotomisés ignorait son statut médical en rapport avec les éléments exploités dans l'étude des comorbidités associées. Chez les réopérés sur 56 soit 46,43% des cas, la distribution des réinterventions est très significative du point de vue statistiques par rapport au cancer et à la diathèse hémorragique avec respectivement 50% et 22,22% des cas. Comme le soulignent beaucoup d'auteurs, le terrain parait jouer un rôle considérable dans le pronostic: l'âge avancé, les pathologies associées, l'immunodépression, les défaillances d'organes, la dénutrition [25,26].

**Les caractéristiques de l'opération initiale** : De ces 56 patients compliqués, 51 soit 91,07% avaient été opérés en urgence et cela pour le processus infectieux qui, à lui seul avait couvert 55,36% des cas avec 31 relaparotomies sur 56. Les laparotomies s'accompagnent d'une immunodépression: elles entraineraient une hyperréactivité des lymphocytes T, une réduction de l'expression du complexe majeur d'histocompatibilité de classe II (HLA-DR) à la surface des monocytes et des lymphocytes T, une réduction de l'expression, des intégrines à la surface des polynucléaires neutrophiles (CD11b/CD18) et une réduction des capacités fonctionnelles de PNN (polynucléaires neutrophiles). Ces modifications disent-ils n'ont rien de spécifique aux infections postopératoires mais elles sont à considérer comme un facteur favorisant très probable de l'infection. Une réduction de l'expression de l'antigène HLA-DR à la surface des monocytes et lymphocytes T à la phase postopératoire précoce a été rapporté chez les sujets qui développent une infection ce déficit d'expression est plus profond et compensé de manière plus tardive que chez les sujets non infectés. Ces modifications sont observées à partir de la 24^ème^ heure post-opératoire jusqu'à 7 jours avant le diagnostic d'infection une susceptibilité individuelle particulière à l'infection et à une réponse inflammatoire variable en fonction des individus pourraient expliquer ces résultats contrastés [27,28].

Enfin la compétence des cellules phagocytaires au cours du sepsis est également à prendre en compte car dans un groupe de patients atteints d'une infection intra abdominale, la réponse oxydative et les propriétés de déplacement des PNN (polynucléaires neutrophiles) étaient altérées [29]. A ces aspects évoqués s'ajoutent les limitations de l'efficacité du traitement antibiotique et le choix du traitement antibiotique. Les concentrations bactériennes élevées favoriseraient la survenue d'un effet inoculum (inactivation des antibiotiques parallèlement à l'accroissement de la population bactérienne). Les ß-lactamines y sont particulièrement sensibles. Les conditions locales d'acidose ou d'hypoxie, la présence de corps étrangers et de débris cellulaires réduisent l'activité de certains antibiotiques comme les Amino glycosides. La production d'enzymes inactivant les antibiotiques (particulièrement les ß-lactamines et les Aminoglycosides) par les bactéries présentes dans le liquide péritonéal limite l'efficacité du traitement. Le geste chirurgical et le lavage abondant de la cavité résolvent ou améliorent largement les deux éléments précédant. Au moment de la reprise chirurgicale 100 patients atteints de PPO généralisées avaient 54 d'entre eux qui étaient porteurs d'au moins une souche bactérienne résistante au traitement empirique administré. Par rapport au choix du traitement antibiotique, il doit être diffèrent des traitements préalables du fait du risque d'émergence des germes résistants [30].

Les cibles préférentielles de ce traitement sont les entérobactéries, les cocci gram positifs et les levures [30]. L'étude de l'antibiothérapie et celle de la septicité du tube digestif (en fonction du siège perforé) peuvent aider à connaitre les germes ciblés et l'antibiothérapie adaptée aux germes ciblés pour être couplé à une chirurgie bien conduite. Les variables n'avaient pas été retenues pour notre série. Dans la présente étude 34 patients sur 56 compliqués avaient été opérés par un chirurgien non qualifié ceci représente 60,71% des cas. Une telle étude examinant la qualité des opérateurs n'a pas été retrouvée dans la littérature. L'organisation de la chirurgie peut être différente. Le bistouri ne se trouve pas dans toutes les mains pour des raisons variées. Les raisons pour lesquelles un médecin en formation, un médecin non chirurgien ou une personne non médecin pratique des interventions chirurgicales dépendent du lieu où l'on se trouve et de la législation de la profession. Dans les milieux spécialisés universitaires la comparaison entre les différentes catégories des chirurgiens peut être biaisée car les laparotomies requérant la main professorale ou celle du chef de travaux ne sont pas les mêmes que celles confiées aux apprentis du premier ou du second mandat qui peuvent d'ailleurs recourir aux « ainés » en cas de problèmes et ces ainés deviennent alors les opérateurs principaux de ces cas.

**Les signes cliniques d'alerte**: Les symptômes et signes étaient nombreux présentés au TAB. 3 Ils n'interviennent pas de la même façon et varient selon le syndrome présomptif tant en ce qui concerne les symptômes généraux, les signes physiques locaux que l'impact de chacun d'eux dans l'indication opératoire. En considérant seulement les trois tableaux cliniques de présomption chirurgicale (péritonite, occlusion et hémorragie) on peut noter à ce TAB. III dans l'ordre décroissant les 6 paramètres les plus représentatifs qui ont été: la distension abdominale croissante mesurée au niveau de l'ombilic, les malaises généraux, la douleur abdominale diffuse et provoquée, la douleur abdominale diffuse et permanente, le débit urinaire diminué, les pansements (de la plaie centrale et/ou des drains) souillés constamment. Quand les six signes se retrouvent associés l'indication de la réintervention s'impose.

## Conclusion

A l'issue de cette étude, il ressort que les relaparotomies sont courantes dans notre milieu (aux hôpitaux universitaires du HAUT KATANGA), elles sont émaillées d'une lourde mortalité. Le retard à poser le diagnostic aggrave le pronostic.

### Etat des connaissances actuelles sur le sujet

- On doit reprendre le plus rapidement possible le laparotomisé qui se complique;
- Un bilan préalable est requis car il faut un faisceau d'arguments en faveur de la reprise;
- Et parfois on dit que mieux vaut reprendre pour rien et à temps plutôt que faire une autopsie.

### Contribution de notre étude à la connaissance

- La chirurgie abdominale est courante chez nous;
- Les complications qui accompagnent cette chirurgie nécessitent parfois la réopération;
- Dans le contexte de pauvreté(le malade ne sait pas se prendre en charge seul et doit être assisté par la famille qui parfois n'a rien) et l'opéré abdominal meurt dans un tableau de défaillance viscérale car il est réopéré en retard.

## Conflits d’intérêts

Les auteurs ne déclarent aucun conflit d'intérêts.
